# Diseases of the Heart and Circulation

**Published:** 1904-08-06

**Authors:** 


*(To be continued.)*

### DISEASES OF THE HEART AND CIRCULATION.

**Primary Non-congenital Disease of the Right Heart.**—Syers[1] states that it is not unusual for a middle-aged patient to present himself complaining of shortness of breath and dropsy, and in whom the heart action is regular and there is no evidence of valvular disease. The symptoms are generally of insidious onset. In some cases there may be moist sounds at the lung bases, but generally they are quite clear. As a rule, the quanity of urine is quite up to the average, and it hardly ever contains albumen. The pulse is usually regular and of good volume, and there may be some hypertrophy of the left ventricle. In some cases the heart dulness is increased to the right, but usually this is not the case. The most obvious explanation of these cases would be dilatation of the right heart from chronic bronchitis and emphysema, but in the cases here considered the history and physical signs negative such a theory. The most probable view is that the right side of the heart is the seat of degenerative changes, the left side being relatively intact. Such a patient, when first seen, may present most of the features of advanced mitral disease, with the important exceptions that the action of the heart is regular, there is no bruit, and the quality of the pulse is good. In treating such cases a combination of digitalis and caffein is of very great service. This, with rest, will often restore a patient, for a time at least, to comparative health.

**Cardiac Dulness in Cancer.**—Gordon[2] states that reduction or loss of cardiac dulness is common in cases of cancer, especially in cases of abdominal cancer towards their end. It may precede cachexia, marked wasting, and loss of skin elasticity, but seems absent in the earliest stages. When present in a doubtful case the sign is very suggestive of the existence of a cancer, and if absent in a doubtful case of considerable duration it is slightly suggestive that the case is not one of cancer. In assessing the value of this physical sign it is of course necessary to exclude the existence of all ordinary causes of alteration in the area of cardiac dulness. The explanation appears to be that the margins of the lungs extend across the front of the heart owing to a loss of elasticity similar to the loss of elasticity of the abdominal skin that occurs in many cases of abdominal cancer.

**Diastolic Murmurs.**—Cabot and Locke[3] record cases in which diastolic murmurs at the aortic and pulmonary orifices occurred without lesion of the valves. They conclude that such murmurs are not uncommon in connection with dilatation of the aorta, localised or diffused. When the pleura and pericardium are adherent, owing to tuberculosis or other causes, diastolic murmurs are occasionally audible. They are notably affected by respiration and by position, and are probably due to suction or pulsion exerted by the heart upon portions of the lung adherent to the pericardium. In cases of intense anæmia one occasionally hears diastolic murmurs, not to be explained by permanent dilatation of the aortic ring, nor as cardio-respiratory murmurs, and not due to a diastolic accentuation of a venous hum. The cause of these murmurs is obscure. Gibson[4] has also recorded some cases of diastolic aortic murmurs without valvular lesions, and in which there was no permanent stretching of the aortic orifice. He thinks that the cause must have been a faulty application of the individual cusps to each other. Similar cases are published by Hamilton and Byers.[5]

**The Cardiosplanchnic phenomenon** has recently been described by Adams.[6] Three factors enter into consideration in the mechanism of blood-supply to the splanchnic vessels—the contraction of the abdominal vessels, respiration, and the regulating vaso-motor action of the nerves. If the abdominal walls become flaccid, blood tends to gravitate into the splanchnic veins and there is a tendency to syncope. Thus the removal of stays in women may induce a feeling of faintness, and the same symptoms may occur when a large quantity of ascitic fluid is removed, and in susceptible individuals when the bladder is emptied or fæces discharged. Every time the diaphragm descends, the intra-abdominal vessels are compressed and the blood is squeezed into the right heart; also the pulmonary vessels expand with each inspiration and suck the blood out of the right heart. If the lower sternal region be percussed in the standing and then in the recumbent position, the note from being resonant becomes dull or flat. This is the cardiosplanchnic phenomenon. Vigorous compression of the abdomen will exaggerate it; on the other hand a few forced inspirations will abolish the dulness. The statements above afford an explanation of the phenomenon—it is due to a filling or over-filling of the right ventricle with blood on recumbency. The elicitation of the cardiosplanchnic phenomenon would prove valuable in the differential diagnosis of a dilated heart from a pericardial exudate; in the latter affection it would not be elicited. Idiopathic syncope and vertigo and the vertiginous attacks of Glénard's disease may be attributed to a defective splanchnic vasomotor mechanism; in such conditions the cardiosplanchnic phenomenon is exaggerated. The predominance of dyspnœic attacks at night in cardio-respiratory affections can be explained by the augmented blood-supply to the right ventricle, the mere result of recumbency. The upright position instinctively assumed in orthopnœa is due, not only to the fact that the extraordinary muscles of respiration may work to better advantage, but also to the additional reason that the blood from the right heart is enabled to gravitate to the splanchnic area. In syncope the object to be achieved is the determination of blood, not so much to the anæmic brain as to the heart. This may be done by compression of the abdomen, either intermittently or by a firm cushion applied by a bandage.

**Exercise in Heart Disease.**—Davis[7] enumerates, as possible methods, massage, Swedish gymnastics, mountain climbing, and resistance gymnastics. Drugs, as digitalis, may temporarily contract the heart when dilated, but permanent recovery of strength can only be obtained by improving the nutrition of the heart and by relieving it of excess of work. Both results are obtainable by judicious exercise of the voluntary muscles. The first effect of muscular work is to increase blood-pressure, but this is followed by a more persistent fall due to dilatation of the arterioles. The primary increase is in proportion to the degree of exertion, the secondary fall is more dependent on the duration of the movements and the size and number of the muscles used. In prescribing exercises the first rule should therefore be that the muscular effort should be slight, but numerous large muscles must be used. Respiration should be deepened but not hurried. The exercises should be graduated, at first only the lightest should be used, and subsequently they should be increased in vigour. Massage and resistance gymnastics are the feeblest forms of exercise, and should be first used in all cases.

1 Treatment, March. 2 Lancet, March 12. 3 Med. Chron., Dec. 4 Edin. Med. Jour. 5 Amer. Jour. Med. Sc., Oct. 6 Amer. Jour. Med. Sc., Jan. 7 Jour. Amer. Med. Assoc., Nov. 14.

